# Editorial for Special Issue “Piezoelectric Aluminium Scandium Nitride (AlScN) Thin Films: Material Development and Applications in Microdevices”

**DOI:** 10.3390/mi14051067

**Published:** 2023-05-18

**Authors:** Agnė Žukauskaitė

**Affiliations:** 1 Fraunhofer Institute for Organic Electronics, Electron Beam and Plasma Technology FEP, 01277 Dresden, Germany; agne.zukauskaite@tu-dresden.de; 2 Institute of Solid State Electronics (IFE), Faculty of Electrical and Computer Engineering, TU Dresden, 01062 Dresden, Germany

The enhanced piezoelectric properties of aluminum scandium nitride (Al_1−x_Sc_x_N or AlScN) were discovered in 2009 by Morito Akiyama’s team [1]. By introducing Sc into wurtzite AlN, the piezoelectric coefficient and electromechanical coupling increase remarkably due to a strong change in the response of the internal atomic coordinates to strain [2]. This had a significant and immediate impact on the 6G RF filter community as well as other fields, where AlN thin films were being developed and applied due to their CMOS compatibility, good mechanical properties, high-temperature stability, and other attractive properties. Before AlScN, the low electromechanical coupling was a barrier to commercializing AlN-based acoustic devices and having a group-III nitride that could compete with other, more established oxide-based piezoelectric materials. To this day, the number of papers on AlScN and other Al-X-N ternary nitrides grows every year, indicating a continuous interest in both academia and industry to advance the technology. In 2019, Simon Fichtner demonstrated that AlScN also has ferroelectric properties, expanding the application field for this fascinating material even further [3]. However, due to the metastability of this ternary nitride [4], there are many unanswered questions about the true limits of AlScN in material synthesis, fundamental properties, and device performance. This Special Issue features thirteen research papers that focus on recent AlScN development and explores the following aspects: growth of AlScN thin films by magnetron sputtering (three papers), fundamental investigations of material properties (four papers), fabrication and performance of AlScN-based ferroelectric and electroacoustic devices (six papers).

The first three papers [5,6,7] address both the growth process optimization and high rate, large area deposition on commercial and industry-relevant substrates at Sc concentration x ≈ 0.27–0.3, which is currently regarded by industry and academia as a reasonable trade-off point, allowing enhanced device performance without suffering too much from metastability-related issues, such as phase separation, an uncontrollable amount of abnormally oriented grains (AOGs), and elemental segregation into Sc-rich and Al-rich domains. Pirro et al. [5] utilize RF bias on the substrate to better understand the relationship between stress levels and the dielectric and ferroelectric properties of the films. Tuning the RF bias resulted in 500 MPa tensile and −2 GPa compressive stress. Films with tensile stress demonstrated better ferroelectric performance and lower losses, whereas high compressive stresses were detrimental to the dielectric and ferroelectric performance of structured metal–ferroelectric–metal (MFM) structures. Su et al. [6] aim to demonstrate a substrate-independent method for depositing high-quality AlScN on various non-metallic substrates using a 20 nm thick AlN seed layer to minimize the number of AOGs. It was postulated that because AlScN is more sensitive to the substrate texture than AlN, focusing efforts on providing a good AlN seed layer rather than process optimization for AlScN on each specific substrate itself is a more applicable approach where AlScN on many different substrates is required for different applications. It was also shown that the addition of an AlN seed layer has only a minor effect on the piezoelectric performance of thick AlScN films. Barth et al. [7] demonstrate AlScN sputtering at a very high deposition rate of 200 nm/min and investigate the homogeneity of structural and piezoelectric properties as well as how they are affected by different growth parameters. In particular, a hybrid unipolar-bipolar pulse mode with an optimized ratio of S_Unipolar_ = 90% was shown to aid in the formation of highly uniform films with good piezoelectric and ferroelectric performance.

In the second section of the Special Issue, four papers [8,9,10,11] focus on investigating the fundamental properties of AlScN, such as the effects of temperature and Sc concentration, and the extraction of device-relevant acoustic parameters. In the in-depth Raman spectroscopy study by Solonenko et al. [8], peak broadening and various mechanisms that contribute to it are investigated. The Raman spectra of AlScN can exhibit up to eight bands, and previously unreported bands could be attributed to second-order phonon modes. In addition, temperature-dependent Raman measurements were performed to demonstrate that the temperature coefficient is a function of not only the Sc concentration but also the defect density. In the study by Wolff et al. [9], in situ annealing up to 1000 °C is combined with X-ray diffraction, and an unexpected volume expansion is observed above 550 °C in AlScN thin films prepared by magnetron sputter epitaxy. It was shown that the transition from linear to non-linear thermal expansion occurs at lower temperatures for higher Sc concentrations and that annealing induces irreversible changes in lattice parameters after cooling down. Intrinsic and extrinsic contributions related to oxygen impurities were separated and carefully explained. Continuing the theme of thermal stability and coupling it with the ferroelectric properties of AlScN, Drury et al. [10] explore the possibility of using this material in high-temperature nonvolatile memory applications. The device-relevant behavior of textured AlScN was studied up to 400 °C. While steady polarization retention was observed even at elevated temperatures, P–E loops showed a decrease in coercive field as a function of temperature, and leakage current density increased significantly at elevated temperatures. Fatigue testing indicated degraded performance above 200 °C; however, at lower temperatures, >10^5^ cycles could be achieved before failure, indicating a high potential for AlScN-based memory applications where ~10^3^ cycles are expected. Next, the capabilities of the non-destructive laser ultrasonics technique are demonstrated by Meyer et al. [11] for the extraction of elastic constants of AlScN required for accurate device design. A special rotating stage was used to investigate the anisotropy of epitaxial c-plane and a-plane AlScN thin films. The latter were especially valuable due to the low amount of curvature in the dispersion curves. As a result, sensitivity analysis indicated that elastic constants c_33_ and c_13_ were the main contributors to the dispersion. Very good agreement with theoretical predictions was achieved, validating this versatile characterization method, especially for anisotropic films/substrate systems.

In the last section, six papers address the fabrication and performance of various AlScN-based devices [12,13,14,15,16,17]. It has been recognized quite early in the development of AlScN that the etching rate drops dramatically compared to pure AlN, and the existing etching approaches used for group-III nitrides must be revised, especially when vertical side-wall geometry has to be well controlled. Wet-etching with KOH is the main focus of the investigation by Tang et al. [12]. In addition to a very comprehensive review of previous dry- and wet-etching studies, both vertical and lateral etch rates are studied systematically as a function of Sc concentration as well as KOH temperature and concentration. A linearity check confirmed etch to be reaction-limited, matching AlN in KOH behavior. However, in the case of vertical etch rate, an expected gradual decrease was observed as a function of Sc. Lateral etching is a highly anisotropic process, and the rate experiences a transition after reaching the lowest point at Sc x = 0.125. The authors were able to demonstrate vertical wall formation at this Sc concentration by exposing the specific planes of AlScN to KOH, which could benefit lamb wave resonators or similar released structures in the future. In a device, it is not only the functional layer that contributes to the overall performance, as shown in the study by Nie et al. [13], where the ferroelectric properties of AlScN are investigated on different metals used for bottom electrodes. In samples with a Mo bottom electrode, a larger polarization loss was observed compared to those where Pt was used. The study indicates that while the formation of abnormally oriented grains is different and has an influence on the ferroelectric performance of AlScN, the inherent contact barrier is also a factor that needs to be considered in device fabrication. Stress control is another very important aspect of electroacoustic device fabrication, as they often contain released structures, such as membranes or cantilevers. Beaucejour et al. [14] demonstrate a stress-compensated growth process allowing low out-of-plane bending in cantilevers. First, the compressive-to-tensile stress gradient was determined as a function of AlScN film thickness, and the average film stress was determined as a function of N_2_ flow. Using this information, the authors were able to produce AlScN thin films where N_2_ flow varied during the growth to compensate for stress. In the best-performing compensated film, this allowed cantilever bending to be reduced from >100 µm to less than 3 µm. The theme of deflection continues in the next study, where AlN and AlScN-based micromirrors are fabricated by Stoeckel et al. [15]. Footprint MOEMS of 2 × 3 mm^2^ and 4 × 6 mm^2^ were designed, with geometrical parameters adjusted based on different mechanical properties of AlScN. A 10-fold increase in the deflection per electric field in AlScN micromirrors was observed compared to AlN, showing the high potential of such MOEMS for future micro-optics applications with large static scan angles. The paper by Lozano et al. [16] focuses on AlScN-on-diamond surface acoustic wave (SAW) resonators and filters. Single crystal and polycrystalline diamond were compared, and in both types of resonators, Rayleigh and Sezawa modes were excited. Although the frequency, acoustic velocity, and electromechanical coupling were similar in both, the quality factor and figure of merit (FOM) were much higher, and insertion loss improved when using the single crystal diamond as a substrate. Finally, dual-mode Lamb wave resonators are demonstrated by Rassay et al. [17]. Patterned molybdenum was used as a bottom electrode, where tapering enabled crack-free overgrowth by AlScN. Dry-etching was used for membrane release. By taking advantage of the ferroelectric properties of AlScN, the authors designed and fabricated resonators that can be periodically poled using an interdigitated transducer (IDT), thus enabling the reversible switching of frequency to cover two modes of operation—0.45–1.6 GHz and 0.8–3 GHz. This novel concept has great potential for single-chip, multi-band devices for modern wireless systems.

To summarize, this Special Issue was very successful in covering all of the main aspects of AlScN research—growth, fundamental and application-relevant properties, device fabrication and characterization. We can see that AlScN technology is mature enough to demonstrate wafer-level material development and complicated devices, but there is still much to discover in terms of deposition process control, anisotropy, and, in particular, ferroelectric behavior. AlScN research is ongoing in a number of research groups around the world, with many more discoveries to come. On behalf of the journal, the Guest Editor, Dr. Agnė Žukauskaitė, would like to thank all of the contributing authors once more and is eager to hear about future work in this field.
